# Depression and anxiety disorders in chronic hemodialysis patients

**DOI:** 10.1192/j.eurpsy.2021.633

**Published:** 2021-08-13

**Authors:** W. Bouali, R. Omezzine Gniwa, R. Ben Soussia, A. Hadj Mohamed, L. Zarrouk

**Affiliations:** Department Of Psychiatry, University Hospital Of Mahdia, Tunisia., Psychiatry, Mahdia, Tunisia

**Keywords:** Depression, Anxiety, Hemodialysis, Associated factors

## Abstract

**Introduction:**

Depression and anxiety are among the most common comorbid illnesses in people with end-stage renal disease. They are under-recognized in hemodialysis (HD) patients.

**Objectives:**

The aim of this study was to assess the prevalence of depression and anxiety disorders among HD patients and its associated factors.

**Methods:**

A cross-sectional study including patients on hemodialysis at the dialysis unit of the University Medical Center of Mahdia, Tunisia, conducted from December 2016 to January 2017. A standard self-administered questionnaire-the Hospital Anxiety and Depression Scale (HADS) was used in the study to measure the presence and severity of anxiety and depression in the study population.

**Results:**

were collated from 55 patients. Overall, 32.7% of patients reported depression and 23.6% reported anxiety. Among symptoms, depression had a significant correlation with diabetes, high blood pressure, and duration of dialysis (p<0.05). Regarding anxiety, this significant correlation was only seen with the duration of dialysis.

**Conclusions:**

The current study showed that the prevalence of depression and anxiety in hemodialysis patients is important and correlates with clinical variables, so effective interventions for mental health should be taken into consideration and the impact of these interventions should be investigated.

